# Nanostructural Influence on Optical and Thermal Properties of Butterfly Wing Scales Across Forest Vertical Strata

**DOI:** 10.3390/ma17205084

**Published:** 2024-10-18

**Authors:** Queenny K. López, Rafael E. Cárdenas, Francisco Ramírez Castro, Karla Vizuete, María F. Checa, César Costa Vera

**Affiliations:** 1 Mass Spectrometry and Optical Spectroscopy Group, Departamento de Fisica, Escuela Politécnica Nacional, Ladrón de Guevara E11-253, Quito 170525, Ecuador; dfrancisco.ramirez88@gmail.com; 2 Museo de Zoología QCAZ, Laboratorio de Entomología, Escuela de Ciencias Biológicas, Pontificia Universidad Católica del Ecuador, Avenida 12 de Octubre 1076 y Roca, Quito 170525, Ecuador; recardenasm@yahoo.com (R.E.C.); mfcheca@puce.edu.ec (M.F.C.); 3 Centro de Nanociencia y Nanotecnología, Universidad de las Fuerzas Armadas ESPE, Gral. Rumiñahui s/n, Sangolquí 171103, Ecuador; ksvizuete@espe.edu.ec

**Keywords:** diffuse reflectance, flight preference, spectroscopy, thermogravimetry, tropical forest, vertical stratification

## Abstract

Butterfly wing scales feature complex nanostructures that influence wing coloration and various mechanical and optical properties. This configuration plays a key role in ecological interactions, flight conditions, and thermoregulation, facilitated by interactions with environmental electromagnetic energy. In tropical forests, butterflies occupy distinct vertical habitats, experiencing significant light and temperature variations. While wing nanostructures have been widely studied, their variation across different vertical flight preferences remains underexplored. This study investigates the wing nanostructures of 12 tropical butterfly species from the Nymphalidae family, focusing on their optical, morphological, and thermal properties across different forest strata. We analyzed the optical response through diffuse reflectance in the UV, Vis, and NIR ranges, correlating these findings with nanostructural configuration and thermal stability using thermogravimetric analysis (TGA). Our results reveal a significant correlation between flight stratification and wing optical responses, alongside distinct nanostructural features within each stratum. This study demonstrates the variability in butterfly wing nanostructures along the vertical stratification of the forest to cope with environmental conditions, raising new questions for future research on eco-evolutionary flight and thermal adaptations. Additionally, this underscores the importance of understanding how these structural adaptations influence butterfly interactions with their environment and their evolutionary success across different forest strata.

## 1. Introduction

Butterflies (Insecta: Lepidoptera) are one of the most successful terrestrial groups with high species richness, local abundance [1,2], and complex wing scales nanostructures [3]. Butterfly wings are remarkable examples of biological nanoengineering. The intricate nanostructures that make up individual scales of chitin and air in three-dimensional arrangements [4,5,6] present characteristic properties that have served as inspiration for bioinnovations, with broad implications for engineering, materials science, and technology [7,8,9].

Nanostructural configuration and cuticular pigmentation are responsible for wing coloration [10,11]. However, physical coloration absorbs more energy at different wavelengths [12], even more than chemical coloration [13]. Together, they are responsible for various mechanical, thermal, and optical properties, such as absorption, dispersion, and light transmittance [14,15,16,17].

Wing-scale nanostructures play a key factor in ecological interactions [18,19,20,21], highlighting their significance in butterfly ecology and evolution [22,23,24,25].

Concerning thermoregulation, although butterflies achieve this through various biological and behavioral mechanisms, such as selecting specific niches within their habitats, the underlying physical principle involves interaction with electromagnetic energy [1,26,27,28]. Most butterflies need to reach a body temperature of 20–50 °C to initiate flight regardless of the temperature of their surrounding habitat [23,24,25]. The development of specific nanostructures on their wings may be an adaptive response to environmental conditions, allowing them to efficiently utilize available energy. For instance, it has been proven that in warmer environments, both species and individuals exhibit a higher reflectance of light [29].

In tropical forests, butterflies occupy diverse habitats, from the canopy to the understory [29,30,31]. This stratification is characterized by significant variations in relative humidity and vegetation composition, delineating distinct habitats within the forest. However, the vertical stratification of tropical forests is primarily characterized by noteworthy variations in light availability (ranging from 1% in the understory to 100% in the canopy) and temperature (with variations ranging from 4 to 10 °C), as these are the primary factors that condition other abiotic elements and the composition of the ecosystem [32]. These variations are much more pronounced than the climatic gradients observed with changes in latitude and altitude, which typically show a temperature variation of 1 °C for every 100 m of altitude [33] or approximately every 150 km toward the equator from the north [34].

The vertical gradient of the tropical forest refers to the variation in environmental conditions, including light, temperature, humidity, and species composition, from the understory to the canopy. Within this vertical gradient, butterflies exhibit diverse flight preferences and habitat associations shaped by their ecological needs such as foraging, mating, and oviposition [35]. Ref. [36] showed a strong relationship between butterflies’ vertical stratification distribution and the allometric variability of the wings. There is a gap in the literature on whether butterflies’ coloration related to nanostructures follows such a pattern along forest strata, in terms of both evaluation and understanding. Consequently, while previous research has elucidated the nanostructural diversity of butterfly wings and its ecological implications, our understanding of how these nanostructures vary across different flight preferences within the vertical stratum of tropical forests has not been evaluated. 

In this study, the knowledge gap regarding the relationship between butterfly wing nanostructures and their vertical habitat stratification in tropical forests was addressed. Optical responses, nanostructural configurations, and thermal properties of forewings and hindwings from 12 butterfly species spanning three distinct forest strata—canopy, mid-stratum and understory—were examined to provide a detailed description of how these adaptations correspond to different vertical habitats. This investigation enhances the understanding of the functional significance of wing nanostructures in response to varying light conditions and contributes to a broader comprehension of how these complex structures influence and optimize ecological interactions across diverse forest environments. 

## 2. Materials and Methods

### 2.1. Specimen Selection

Twelve species of scavenging butterflies from the family Nymphalidaefrom the Tropical Forest in the biogeographic region of Ecuadorian Chocó (00°28′ S, 79°12′ W) [37,38], were selected. Stratification was determined by [36] using a 0–1 stratification index, where values closer to 0 indicated species flying closer to the ground (Table 1). For this study, the species were divided into three main groups of vertical stratification: canopy, mid-stratum, and understory (four species representative of each stratum). The species were provided by the butterfly collection of the QCAZ Museum of Zoology of the School of Biological Sciences at the Pontifical Catholic University of Ecuador (PUCE).

The collection and storage methods used were described by [37]. For this study, one specimen from each of the twelve species was selected from the QCAZ museum. The wings of each individual were removed using a scalpel and entomological forceps. From the first set of wings (forewing and hindwing), 5 × 5 mm squares were cut from six specific areas for optical (Motic, Inc., Schertz, San Antonio TX 78154, USA) and scanning electron microscopy (SEM) (Tescan Group, Brno-Kohoutovice, Czech Republic) analysis. Additionally, the entire wings of the second set were used for Near-Field Multispectral analysis and thermogravimetric analysis (TGA) (Metler Toledo Inc., Columbus, OH, USA).

### 2.2. Optical Microscopy Imaging

Each area of interest was placed on a coverslip and visualized under a standard trinocular optical microscope (Model B1-253ASC, Motic, Inc., Schertz, San Antonio, TX 78154, USA). Optical and digital photographs of the areas were obtained using 10×, and 40× objectives. Full RGB color images were obtained with a 10 Mp CMOS camera (Moticam 10, Motic, Inc., Schertz, San Antonio, TX 78154, USA).

### 2.3. Scanning Electron Microscopy 

**Figure 1:**
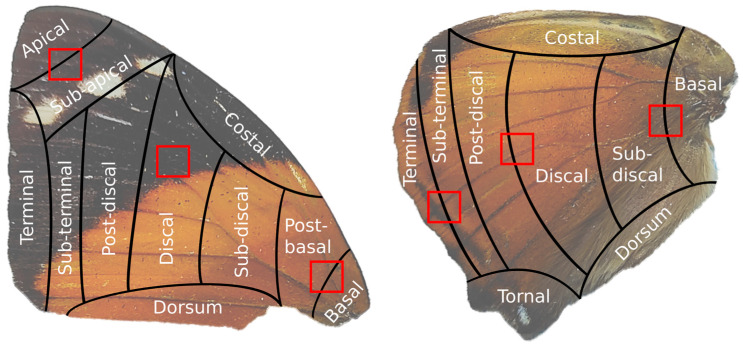
Areas of the wings analyzed spectrally and microscopically represented in red squares.

Electron micrographs (obtained using a scanning electron microscope Tescan Mira 3 equipped with a Schottky-type field emitter, Tescan Group, Brno-Kohoutovice, Czech Republic) were taken from three areas of the forewing and hindwing of each species. These areas were the apical–sub-apical, discal, and basal-post-basal zones for forewings and terminal–sub-terminal, discal–post-discal, and basal–sub-discal zones for hindwings (Figure 1).

The selected parts were fixed to aluminum specimen holders with conductive carbon tape and fixed again with silver tape, exposing only the central area of the section. A 20 nm thick gold coating (99.99% purity) was deposited on the samples (Quorum Q150R ES evaporator, Quorum Tech, Lewes BN8 6BN, UK). 

### 2.4. Near-Field Multispectral Analysis

Multiband spectroscopic analysis was performed with a homemade two-dimensional multispectral scanner (Figure S4), which captures the spectrally decomposed diffuse reflectance of the sample in 10 channels. Each channel corresponds to the reflectance from an individual LED emitter. The spectral range covered was 375–940 nm, including these 10 channels. An additional fluorescence channel can be defined if desired, using UV LED illumination. Measurements were conducted in a dark room with light collected by a collimator-terminated optical fiber at 90° relative to the horizontal plane, while illumination was performed at 45° using a D’Alambertian diffuser common to all LED sources. The collection-illumination device was scanned about the surface of the sample at a distance of 3 mm between the sample and collecting optics. The instrument can measure samples up to a few tens of centimeters with a maximum resolution of 428 µm per pixel. For these measurements, the entire areas of the forewing and hindwing of each of the selected species were scanned, covering areas from 1.5 × 1.5 × 5 × 5 cm^2^ at maximum resolution, depending on the species. The reflectance spectra for each channel were registered using a compact CCD spectrometer (US2000 from Ocean Optics Inc., Dunedin, FL, USA) and comprise 2048 12-bit pixels from 200 to 980 nm with a resolution of ~0.3–10.0 nm FWHM. 

Out of the 10 LED channels, UV, Vis, and NIR indices were established to represent an integrated response across these spectral ranges. The UV index was defined by the 375 nm (±10 nm) LED, and the visible range included the responses from the 435 nm (±15 nm), 475 nm (±20 nm), 525 nm (±22 nm), 590 nm (±10 nm), and 625 nm (±10 nm) LEDs. The NIR index was defined by the 700 nm (±25 nm), 760 nm (±30 nm), 810 nm (±25 nm), and 940 nm (±25 nm) LEDs. These indices were calculated by dividing the summed area of the corresponding reflectance signals by the total area of the three indices together (effectively, all the illuminating LEDs). Consequently, all indices are less than 1 and monotonically define the relative reflectance within these spectral ranges.

Reflectance signals for the 10 LEDs were collected at each scan position (pixel) and referenced to a “perfect” white Teflon^®^ standard. The pixel-by-pixel reconstruction of images from the spectral data and the posterior data processing were performed using Mathematica^®^ (version 12, Wolfram Research Inc., Champaign, IL, USA). This included three selected areas of 25 × 25 mm^2^ per wing, which were aligned with the areas measured using SEM. For each wing area, the relative reflectance indices were calculated (Index_i_ = area_i_/(area_UV_ + area_Vis_ + area_NIR_); i = UV, Vis, and NIR) corresponding to the relative area under the curve of the resulting normalized spectra. These indices were then compared between species and strata.

### 2.5. Thermogravimetry

Thermogravimetric analysis was carried out in 70 µL alumina sample cells (Mettler Toledo Model TGA 2, Metler Toledo Inc., Columbus, OH, USA) for both complete wings of each species under the following conditions: dynamic mode, initial temperature: 25 °C, final temperature: 600 °C, heating rate: 10 °C/min, nitrogen atmosphere at 50 mL/min, balance resolution: 0.1/1 µg, and weighing precision: 0.005%.

### 2.6. Statistical Analysis

The statistical analyses included comparisons between different groups and within the groups for optical response analysis and thermogravimetry. The groups consisted of four butterfly species each, categorized into a “stratum” based on their similar stratification indices. To select the appropriate statistical test, the assumptions of normality and homogeneity of variances in the data were evaluated by applying the Shapiro–Wilk test, *W*, and Levene test, *F*. For parametric data that met the assumptions of normality and homogeneity of variances, ANOVA was applied followed by the Tukey test. If the data did not meet these assumptions, the Kruskal–Wallis test was used, followed by the Dunn test. Additionally, Pearson correlation analysis was performed to examine the relationships between the spectroscopic indices and the stratification indices of each species. Further correlation analyses investigated the pairwise relationships between specific indices: NIR vs. Vis, UV vs. Vis, and NIR vs. UV.

For the statistical comparison of groups, a dendrogram was generated to analyze the optical responses of the species based on their reflectance indices in the NIR, Vis, and UV ranges. To enable a comprehensive comparison of the reflectance characteristics, the three indices—NIR, Vis, and UV—were combined into a single dataset. This approach provides an integrated view of the optical profiles across different species. Hierarchical clustering analysis was performed using Ward’s method, which minimizes the total within-cluster variance at each step, thereby grouping species with similar optical responses. The Euclidean distance was used as the “dissimilarity index” to create a distance matrix from the combined reflectance indices. The dendrogram offers a visual representation of the similarities and relationships among species, revealing patterns and trends in their optical responses relative to different forest strata and light conditions. The threshold for statistical significance in all tests was set at a *p*-value of 0.05. All analyses were performed using Google Colab software (2024).

## 3. Results and Discussion

### 3.1. Large-Field Optical Images 

**Figure 2:**
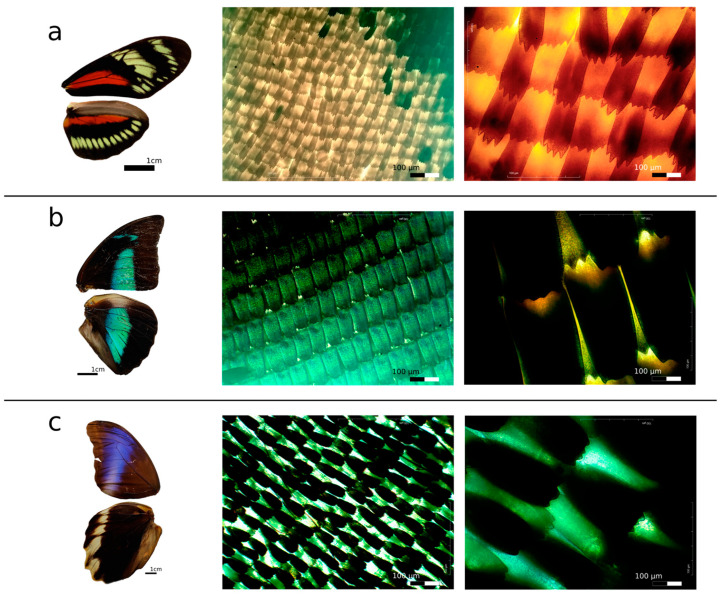
Macrophotographs and optical microscopy stacked images using 10× and 40× objectives: (**a**) *Heliconius hecalesia* (representative of canopy), (**b**) *Archaeoprepona demophon* (representative of mid-stratum), and (**c**) *Caligo atreus* (representative of understory). Additional species analyzed from each stratum are shown in Supplementary Material Figure S1.

The large-field optical images for the three strata show that the scales are arranged like overlapping tiles and the two layers of scales—ground and cover scales—alternate along vertical rows. The wing scales all point toward the proximal end of the wing (away from the body), which is oriented downward in these images. The scales are uniformly elongated, feature longitudinal ridges on the upper lamina, and exhibit “finger-like” projections at their ends (Figure 2 and Figure S1). Additionally, regardless of the strata, all the species present finger-like projections, with their number varying from 0 (flat or rounded) to 8 across the wings, and present differences in their quantity between the ground and cover scales.

### 3.2. Morphological Characterization of Nanostructures

Four types of nanostructures were identified in different parts of the analyzed wings, based on the following criteria: (a) Type I: non-specialized structures, characterized by quasi-parallel ridges along the scale covered by fine lamellae. They have a formation of “windows” made of ribs or transverse ribs that extend from crest to crest, accompanied by nanoribs that in turn form their rows. From these windows between ridges, a series of pillars or “trabeculae” join the lower and upper laminas [9,39,40]. (b) Type II: crest of lamellae. These structures are formed by a series of overlapping lamellae as multilayer ridges that can be parallel or quasi-parallel to the base of the scale, commonly consisting of three to nine lamellae [8,41,42,43]. (c) Type III: porous structures: structures that form when specialized transverse ribs extend from ridge to ridge. Instead of windows, there is a non-regular porous pattern where there may or may not be transverse ribs [8,44]. (d) Type IV: body lamellae. These structures are formed by transverse ribs that extend from crest to crest, covering most of the surface of the scale. The windows usually disappear or only a few of them remain, more so in rows with greater separation [3,9,41].

**Figure 3:**
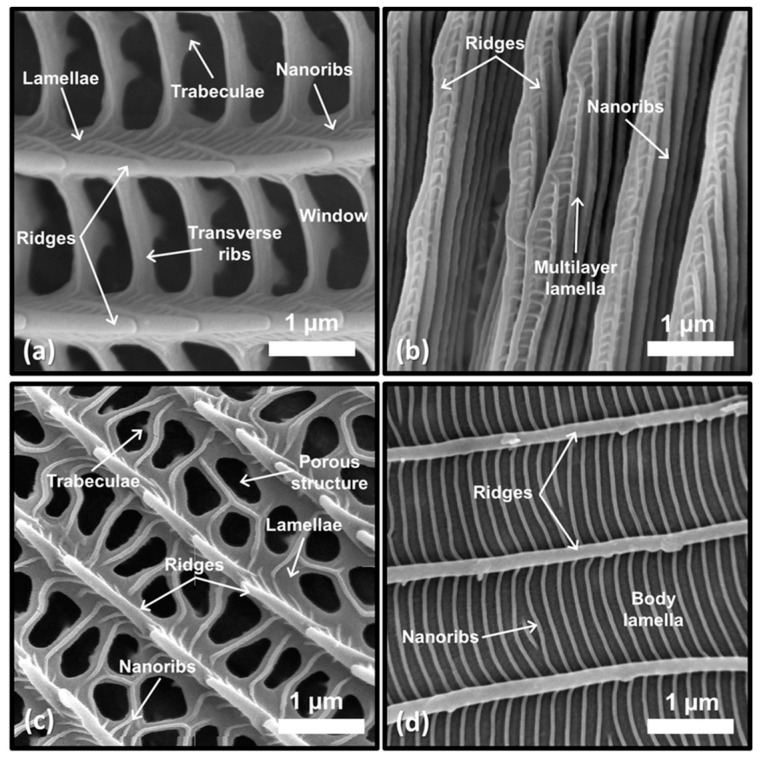
Morphological classification of nanostructures.

Although these structural sequences are periodic, none form a photonic band gap and therefore do not qualify as photonic crystals [45] (Figure 3).

**Figure 4:**
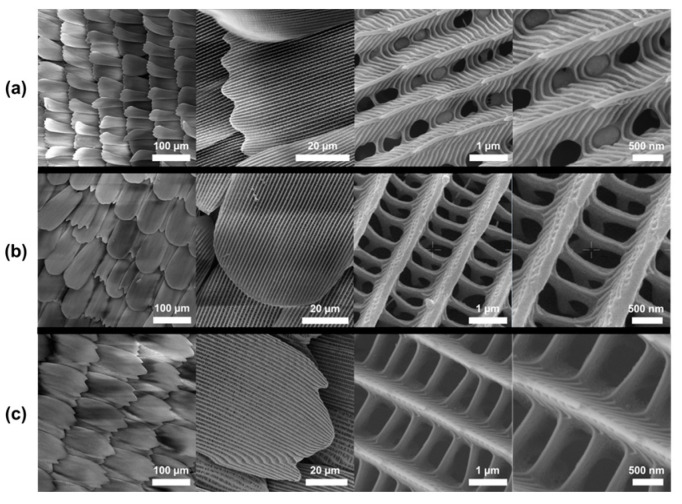
SEM micrographs of the discal area of the forewing at different magnifications of (**a**) *Heliconius hecalesia* (representative of canopy), (**b**) *Archaeoprepona demophon* (representative of mid-stratum), and (**c**) *Caligo atreus* (representative of understory).

Electron micrographs of the forewing and hindwing of the 12 species revealed a combination of different types and quantities of micro- and nanostructures. Although all species exhibited a significant proportion of Type I nanostructures, it was possible to observe that in the canopy species, the ridges are more pronounced and there is a greater predominance of nanoribs, while the understory species present relatively “flatter” structures and morphologies with greater spacing (Figure 4 and Figure S2). Type II nanostructures were only observed in mid-stratum species, while Type III and Type IV morphologies were also present in both canopy and understory species. In addition, most of the observed scales in the species from all strata show an anisotropic configuration formed by long channels forming the ridges of the nanostructures. 

### 3.3. Optical Response

**Figure 5:**
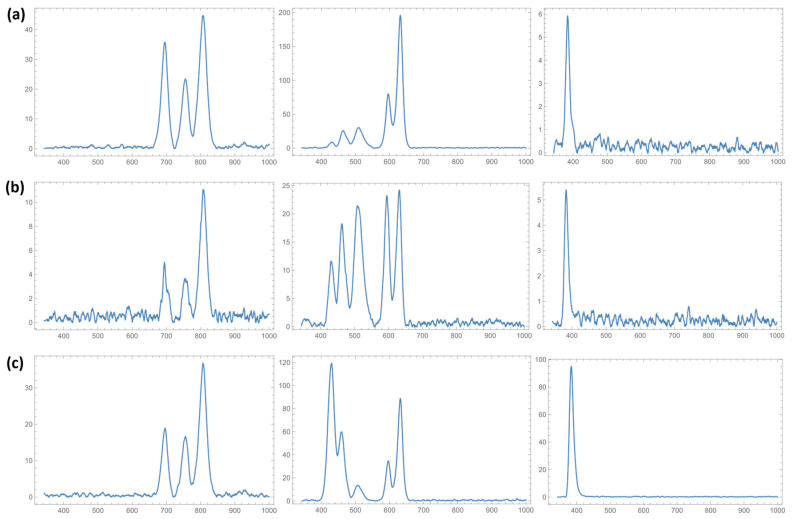
Diffuse reflectance response (I, u.a.) in the NIR, visible, and UV ranges (λ, nm) for (**a**) *Heliconius hecalesia* (representative of canopy), (**b**) *Archaeoprepona demophon* (representative of mid-stratum), and (**c**) *Caligo atreus* (representative of understory).

Diffuse reflectance spectra of the analyzed areas and species were obtained (Figure 5). In all cases, canopy and mid-stratum species showed significantly higher reflectance indices in the visible range and absorbance in the NIR than understory species.

The optical response indicates that the wings, both forewings and hindwings, of the analyzed species exhibit greater diffuse reflectance in the visible range, followed by in the NIR and UV ranges. Although the scales possess diverse nanostructures, most of the morphologies that make up the three-dimensional arrangement (ridges, microribs, cross ribs, lamina, and pores) are associated with the blue color (wavelengths between 450 and 500 nm) [45]. Additionally, the anisotropic arrangement of the scales modifies the polarization of the re-emitted light, creating color contrasts observed in the species and affecting the reflectance of certain ranges, specifically in the blue-to-UV ranges. The ridged structure influences the scattering and reflection of light, enhancing specific wavelengths while reducing others, thus contributing to the vibrant and varying color displays seen across different species and strata [46,47]. It is important to note that the effect of light polarization on the studied structures was not investigated in this study. Consequently, the results presented here are averaged over all possible polarization angles in our results, and this physical variable may have great importance in the final observed response.

**Figure 6:**
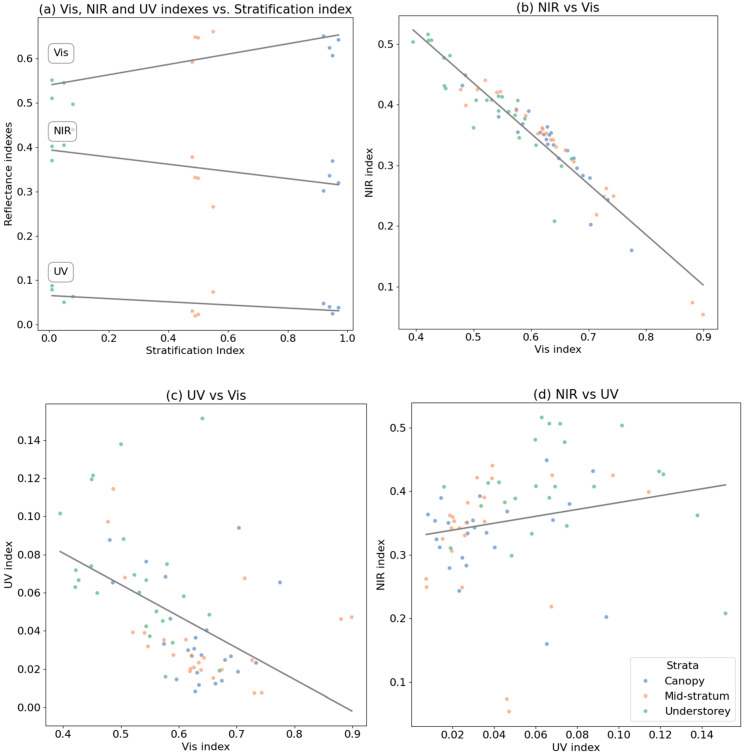
Linear correlations between (**a**) stratification index of the 12 species and the reflectance indices in the visible (R^2^ = 0.6), NIR (R^2^ = 0.41), and UV (R^2^ = 0.36) ranges; (**b**) NIR and Vis indices of forewings and hindwings (R^2^ = 0.9); (**c**) UV and Vis indices of forewings and hindwings (R^2^ = 0.26); and (**d**) NIR and UV indices of forewings and hindwings (R^2^ = 0.04).

Pearson correlation analysis revealed that there was a positive correlation between the stratification index (see Materials and Methods) and the Vis index and a negative correlation with the NIR and UV indices with 95% confidence (*p_NIR_* = 0.024; *p_Vis_* = 0.004; *p_UV_* = 0.039) (Figure 6a). 

Complementarily, for the relationship between NIR and Vis, an R^2^ of 0.9 (Corr: −0.95, *p_NIR-Vis_*: 2.82 × 10^−36^) suggests a very strong inverse linear relationship, meaning that as the Vis index decreases, the NIR index tends to increase significantly, and vice versa (Figure 6b). The relationship between UV and Vis, with an R^2^ of 0.26 (Corr: −0.51, *p_UV-Vis_*: 6.01 × 10^−6^), was also inverse but much weaker, indicating that only a small part of the variation in UV can be explained by changes in Vis, suggesting other influencing factors (Figure 6c). In contrast, the relationship between UV and NIR, with an R^2^ of 0.04 and a direct relationship, shows that there is virtually no linear relationship between these variables, implying that changes in UV and NIR are not significantly correlated (Corr: 0.20, *p_UV-NIR_*: 8.96 × 10^−2^) (Figure 6d). Therefore, this correlation was not statistically significant and could be due to random chance.

**Figure 7:**
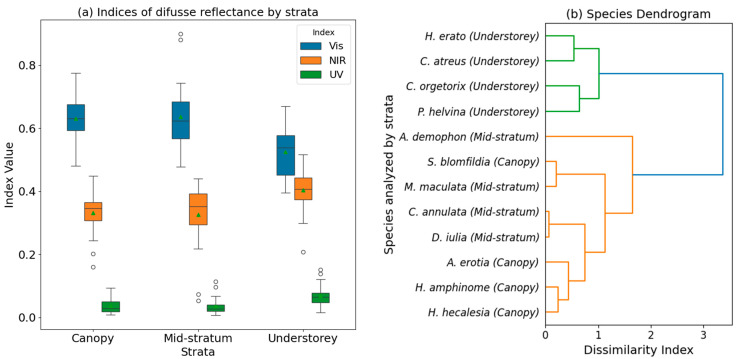
(**a**) Box-and-whisker plots for reflectance data in the NIR, Vis, and UV light ranges according to the forest stratum of the species; (**b**) hierarchical dendrogram of the species based on the combined UV, Vis, and NIR reflectance indices.

Statistical comparison of species groups across different forest strata revealed no significant differences between canopy and mid-stratum species in the three analyzed light ranges. However, both canopy and mid-stratum species exhibited statistically significant differences compared to understory species (Figure 7a).

A Kruskal–Wallis test for equal medians was applied to the reflectance data in the NIR and UV ranges because they did not comply with the assumption of normality (*W*_NIR_ = 0.935, *W*_UV_ = 0.897, *p*-value < 0.05). In both cases, the test had a *p*-value < 0.001, and according to Dunn’s post hoc test, differences occurred between the canopy and mid-stratum NIR and UV datasets compared to the understory datasets. In the case of the visible range data, considering the criteria of normality and homoscedasticity (*W*_Vis_ = 0.975, *F*_Vis_ = 1.32, *p*-value > 0.05), an ANOVA test was applied with a *p*-value < 0.001. Similarly, Tukey’s post hoc analysis showed differences between the canopy and mid-stratum datasets concerning the understory. To jointly analyze the optical response in all light ranges, a dendrogram of the analyzed species was generated based on the reflectance indices and Euclidean distance, allowing the species to be grouped according to their similarities in the reflectance response of the NIR, Vis, and UV indices combined (Figure 7b).

The statistical correlations suggest that the nanostructural adaptations in butterfly wings, which influence their optical properties, are indeed correlated with their habitat preferences within the forest. In comparing species from open habitats with strong lighting (e.g., grasslands) and those from habitats with weak and diffuse lighting (e.g., forests), a variation in the structures responsible for reflecting the blue color as a sexual signal has also been observed. This response is directly related to the light conditions of the surrounding habitat [11], despite the configuration of the wing scales allowing diffuse propagation of light through the structures that compose them [48]. The significant correlation between the NIR and visible indices further emphasizes the coherence of the optical responses across different light ranges, indicating consistent nanostructural adaptations across the spectrum. It was observed that understory species exhibited much lower optical response (i.e., diffuse reflectance) than species from the upper strata at all wavelengths analyzed. The reduced reflectance (and increased absorption of the available solar radiation) in cooler environments may help butterflies attain the necessary body temperatures for flight more quickly [49]. Butterflies that predominantly fly in the canopy reflect more visible light and absorb more in the NIR range, while understory species reflect more in the NIR range and absorb more visible light. Open structures allow light to penetrate deeper before being scattered, leading to less reflection of visible light compared to closed nanostructures. However, these structures may be more efficient at dispersing longer wavelengths, such as in the NIR range. Since the visible-to-NIR wavelengths are important for heat gain via the absorption of incident solar irradiation [50], this differential reflectance is likely an adaptation to their respective light environments. Canopy species exhibited more pronounced nanostructures (i.e., higher ridges and a higher number of ribs) and visually exhibited greater coloration. It has been previously established that ridges and multilayer structures stand out for having the widest color range [45]. Clustering analysis revealed patterns of similarity in the reflectance responses of butterfly species, providing further evidence of the coherence in nanostructural adaptations across the NIR, Vis, and UV spectra, especially when comparing understory species and other strata species. Understory species showed greater UV reflectance than mid-stratum and canopy species, indicating a lower absorbance. This could be related to the limited availability of UV light, as less than 3% of the solar spectrum reaching the Earth’s surface is in this range [51]. Absorption in the UV range may be linked to the presence of proteins such as tyrosine, a precursor of chitin [51], or pigments derived from pterin [52]. Additionally, the architecture of the wing scales significantly modifies the penetration of light with wavelengths ranging from blue to UV. This increased UV reflectance could be an adaptive feature for effectively utilizing the available UV light for purposes such as signaling or protection [11,45]. 

Approximately 37% of the known species in the Nymphalidae family have been studied for coloration. Structures with ridges or lamellae have been identified in 80% of the subfamilies, with these structures primarily observed in the visible and UV ranges [45]. This study contributes to previous findings by not identifying photonic crystals in any of the analyzed areas. The discussion of brightness performance and variability among different nanostructure categories provides insights into the trade-offs between structural complexity and optical properties. However, there are still contradictory opinions and studies regarding the influence and interaction of wing nanostructures with different ranges of the electromagnetic spectrum, where their response and biological importance might extend beyond optical effects to include thermal aspects as well [45,49,50].

### 3.4. Thermogravimetry

**Figure 8:**
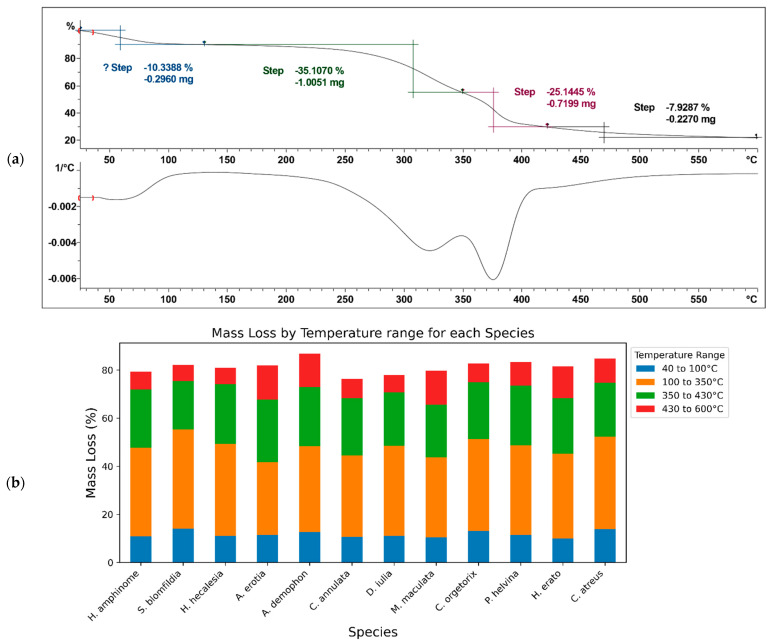
(**a**) Thermogram obtained from fore- and hindwing of each species (complete thermograms, see Figure S3); (**b**) mass loss (%) for each species by temperature range.

The thermal properties of the wings were investigated by thermogravimetric analysis (TGA). Fortunately, despite the delicate nature of the samples, interesting results pointing to specific phase changes were obtained, which might eventually indicate the benefits of such structures for the assimilation of radiant energy in the corresponding strata for the different species studied. The resulting thermograms show mass loss in four main phases corresponding to four temperature ranges (Figure 8a and Figure S3). The first phase (25 to 100 °C) of mass loss is attributed to the evaporation of water present in the wings and encapsulated in the micro- and nanostructures. In this range, a general mass loss of 11.41 ± 1.35% and 12.11 ± 1.74% is observed for the forewings and hindwings, respectively. The species *Heliconius hecalesia* (canopy), *Pierella helvina* (understory), and *Caligo atreus* (understory) show greater mass loss with 14.07%, 13.18%, and 13.82%, respectively. The second phase (100 to 350 °C) shows a mass loss of 35.72 ± 3.78% for the forewing and 36.89 ± 3.11% for the hindwing. The species that register the greatest mass loss are the same as in the first range, with 41.22%, 38.17%, and 38.53%, respectively. The mass loss in this temperature range is attributed to the initial degradation of chitin and probably other less stable compounds, mainly organic molecules such as pigments [53]. The third phase (350 to 430 °C) corresponds to total mass losses of 23.55 ± 2.17% and 23.39 ± 2.15% for the forewings and hindwings, respectively. In this range, the species with the greatest mass loss are *Smyrna blomfildia* (canopy), *Heliconius erato* (understory), and *Adelpha erotia* (canopy) with 24.82%, 26.05%, and 24.91%, respectively. In the final phase (430 to 600 °C), the greatest mass losses corresponds to the species *Smyrna blomfildia* (canopy), *Colobura annulata* (mid-stratum), and *Dryas iulia* (mid-stratum) with losses of 14.24%, 13.97%, and 14.19%, respectively. The overall mass loss is 9.43 ± 4.52% for the forewing and 10.41 ± 4.43% for the hindwing. These phases together are attributed to the breakdown of the polysaccharide structure and the disintegration of acetylated and deacetylated units within the chitin polymer [54,55]. The presence of allomeric molecules, such as β- and γ-chitin, cannot be ruled out [56] (Figure 8b). 

The statistical analysis aimed to determine whether there were significant differences in mass loss across different temperature ranges, forest strata (canopy, mid-stratum, understory), and species. For the data from phases two and three, the Shapiro–Wilk test was used to assess normality, with W-values of 0.959 and 0.965, respectively, indicating that these datasets met the parametric assumptions. The Levene test was applied to evaluate the homogeneity of the variances, yielding F-values of 2.214 and 0.823, respectively. Since the data met the assumptions of normality and homogeneity, parametric tests were conducted. ANOVA was used to compare the mean mass loss between different groups and species. No significant differences were found, as *p*-values were greater than 0.05.

In contrast, the data from phases one and four did not meet parametric assumptions. The Shapiro–Wilk W-values for these phases were 0.914 and 0.729, respectively, and *p*-values were less than 0.05, indicating non-normality. As a result, non-parametric tests were used for these datasets. The Kruskal–Wallis test was employed to assess differences in median mass loss between groups and species. Similarly, no significant differences were observed, with *p*-values greater than 0.05.

The lack of statistically significant differences in mass loss across temperature ranges, species, and forest strata suggests a uniform thermal response and structural composition of butterfly wings. This uniformity highlights the robustness and resilience of butterfly wing nanostructures against thermal degradation, regardless of species variation or habitat preference hinting to a shared ancient evolutionary trait. However, further research is needed to explore the specific molecular composition and structural properties of butterfly wing nanostructures to elucidate their thermal stability and ecological significance.

## 4. Conclusions

The objective of this study was to comprehensively analyze the optical, morphological, and thermal properties of 12 selected tropical butterfly species from the Nymphalidae family distributed along forest stratified compartments (canopy, mid-stratum, and understory). Through examining these properties, the aim was to understand the underlying mechanisms in the phenomenon of interacting with electromagnetic energy and urge further research on potential applications of butterfly wing characteristics.

The findings suggest that the unique optical, morphological, and thermal traits observed in these butterfly species are closely linked to ecological and evolutionary factors. A key ecological aspect is the stratification of flight, where different species occupy distinct vertical layers within their habitats. This stratification influences their wing morphology and optical responses, as each layer presents unique light and temperature conditions and potential ecological pressures.

The variation in optical response among the species studied reflects unique adaptations to different environmental settings. Understory species show significantly lower total reflectance (combining the NIR, visible, and UV response) compared to canopy and mid-stratum species. Butterflies inhabiting higher strata often exhibit brighter patterns as they reflect more of the visible range of light, while those at lower strata may have more subdued coloration due to the reflection of the ranges NIR and UV. Nanostructures with smaller spacing and the presence of voids have a higher reflectance in the visible range and a lower one in the NIR range since there is a strong inverse correlation between the two. These nanostructures are predominantly found in canopy and mid-stratum species.

## Figures and Tables

**Table 1:** Selected species and stratification indices within the forest (taken from Ref. [36]).

Species	Vertical Flight Stratum	Stratification Index
*Hamadryas amphinome*	Canopy	0.97
*Smyrna blomfildia*	Canopy	0.95
*Heliconius hecalesia*	Canopy	0.94
*Adelpha erotia*	Canopy	0.92
*Archaeoprepona demophon*	Mid-stratum	0.55
*Colobura annulata*	Mid-stratum	0.50
*Dryas iulia*	Mid-stratum	0.49
*Manataria maculata*	Mid-stratum	0.48
*Catoblepia orgetorix*	Understory	0.08
*Pierella helvina*	Understory	0.05
*Caligo atreus*	Understory	0.01
*Heliconius erato*	Understory	0.01

## Data Availability

The original data presented in the study of multispectral spectroscopy and TGA are openly available on FigShare at https://doi.org/10.6084/m9.figshare.26510626 and https://doi.org/10.6084/m9.figshare.26516152, respectively.

## Supplementary Materials

The following supporting information can be downloaded at: https://www.mdpi.com/article/10.3390/ma17205084/s1, Figure S1: Macrophotographs and optical microscopy stacked images using 10× and 40× objectives of all analyzed species. Figure S2: SEM micrographs of nanostructures present in wings of analyzed species. Figure S3: Thermograms obtained from forewing and hindwing of analyzed species. Figure S4: Homemade two-dimensional multispectral scanner for multispectral analysis.

## Abbreviations

LED	Light-Emitting Diode
NIR	Near Infrared
SEM	Scanning Electron Microscope
RGB	Red, Green, and Blue
TGA	Thermogravimetric Analysis
UV	Ultraviolet
Vis	Visible
