# Supplementary figures and images for: Nanostructural Influence on Optical and Thermal Properties of Butterfly Wing Scales Across Forest Vertical Strata
Source: Materials (Basel). 2024 Oct 18;17(20):5084. doi: 10.3390/ma17205084 (PMC11509712; doi:10.3390/ma17205084)

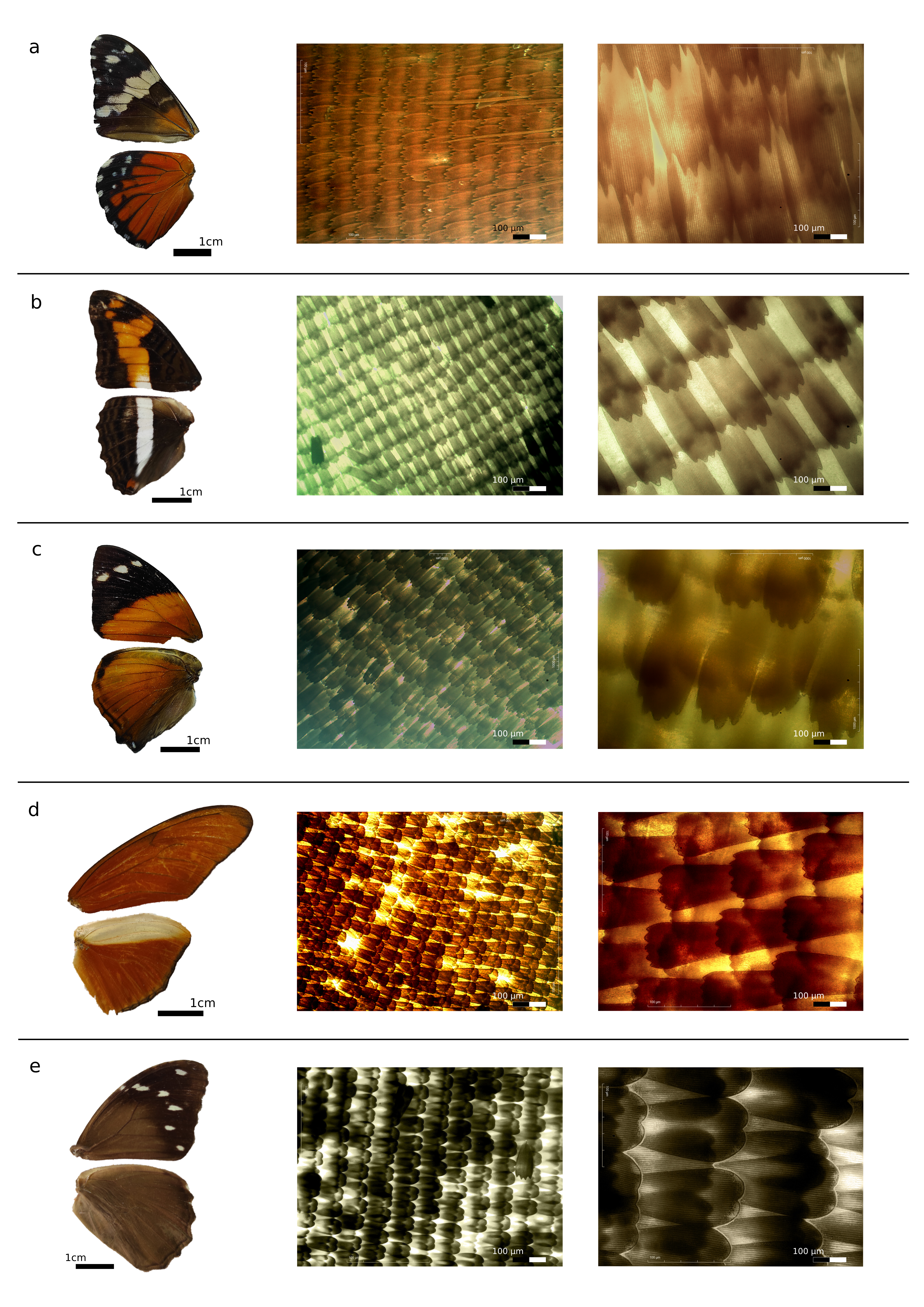

Supplement: Supplementary file 1 [file materials-17-05084-s001.zip › Figure S1.1.tiff]

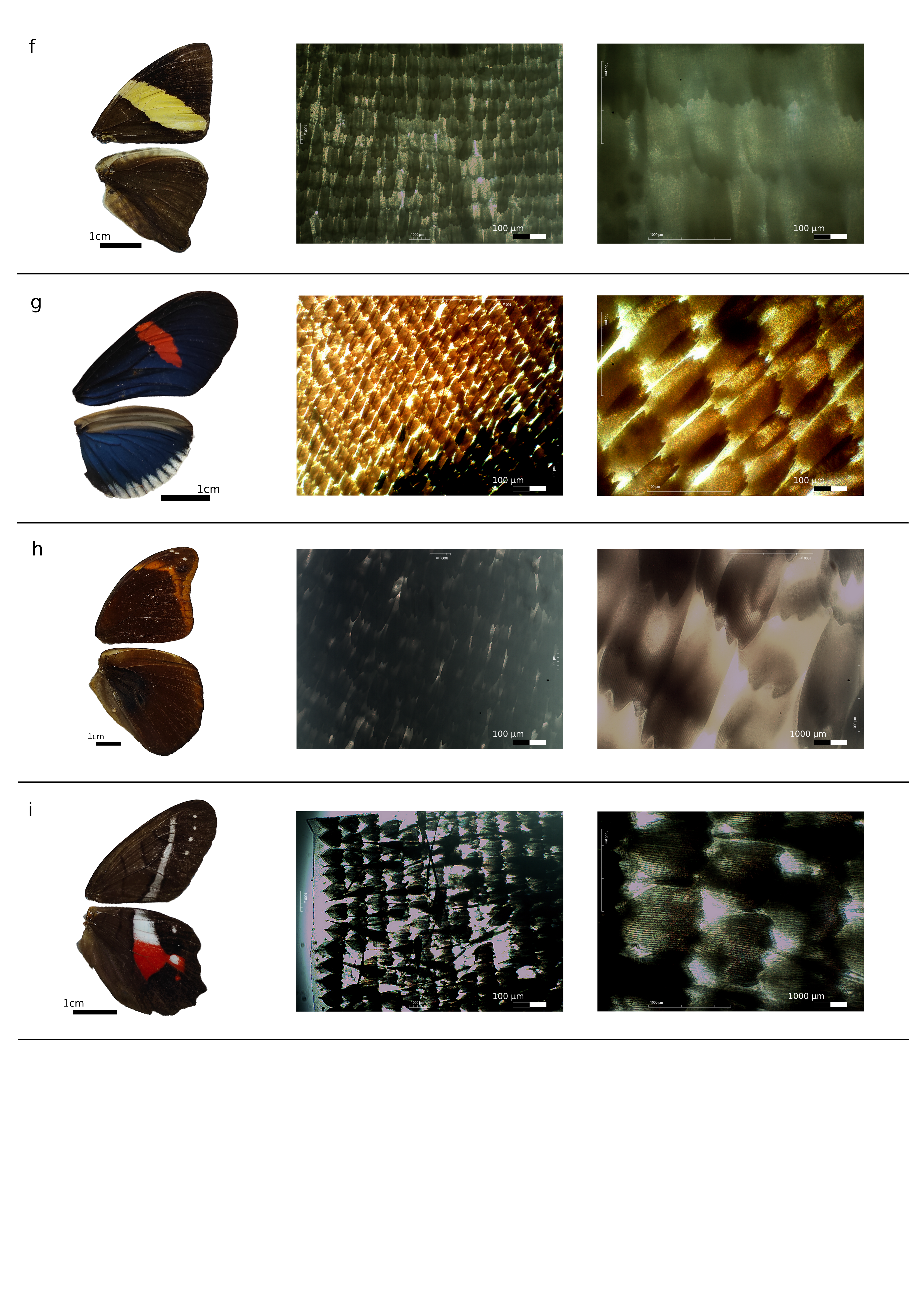

Supplement: Supplementary file 1 [file materials-17-05084-s001.zip › Figure S1.2.tiff]

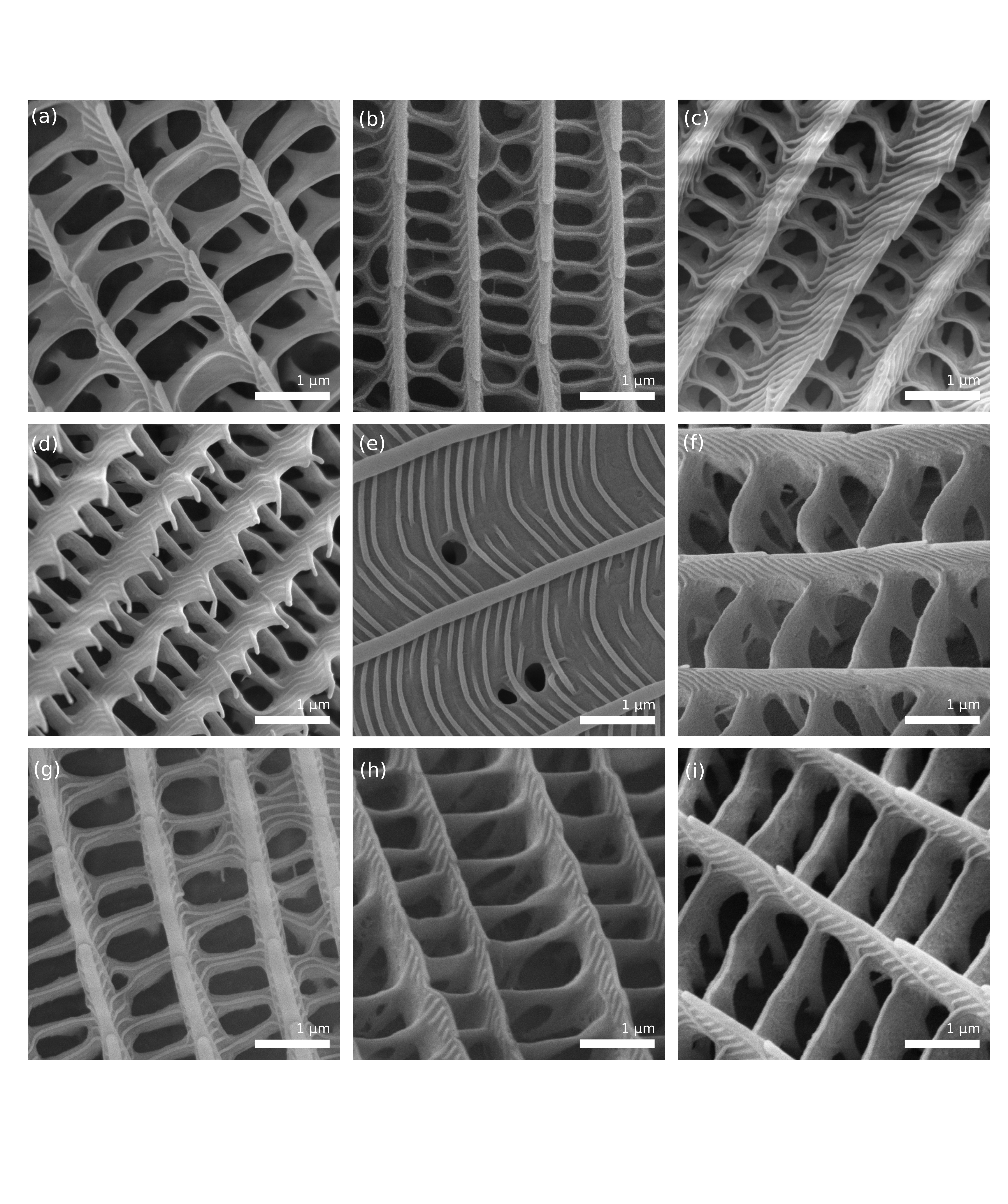

Supplement: Supplementary file 1 [file materials-17-05084-s001.zip › Figure S2.tiff]

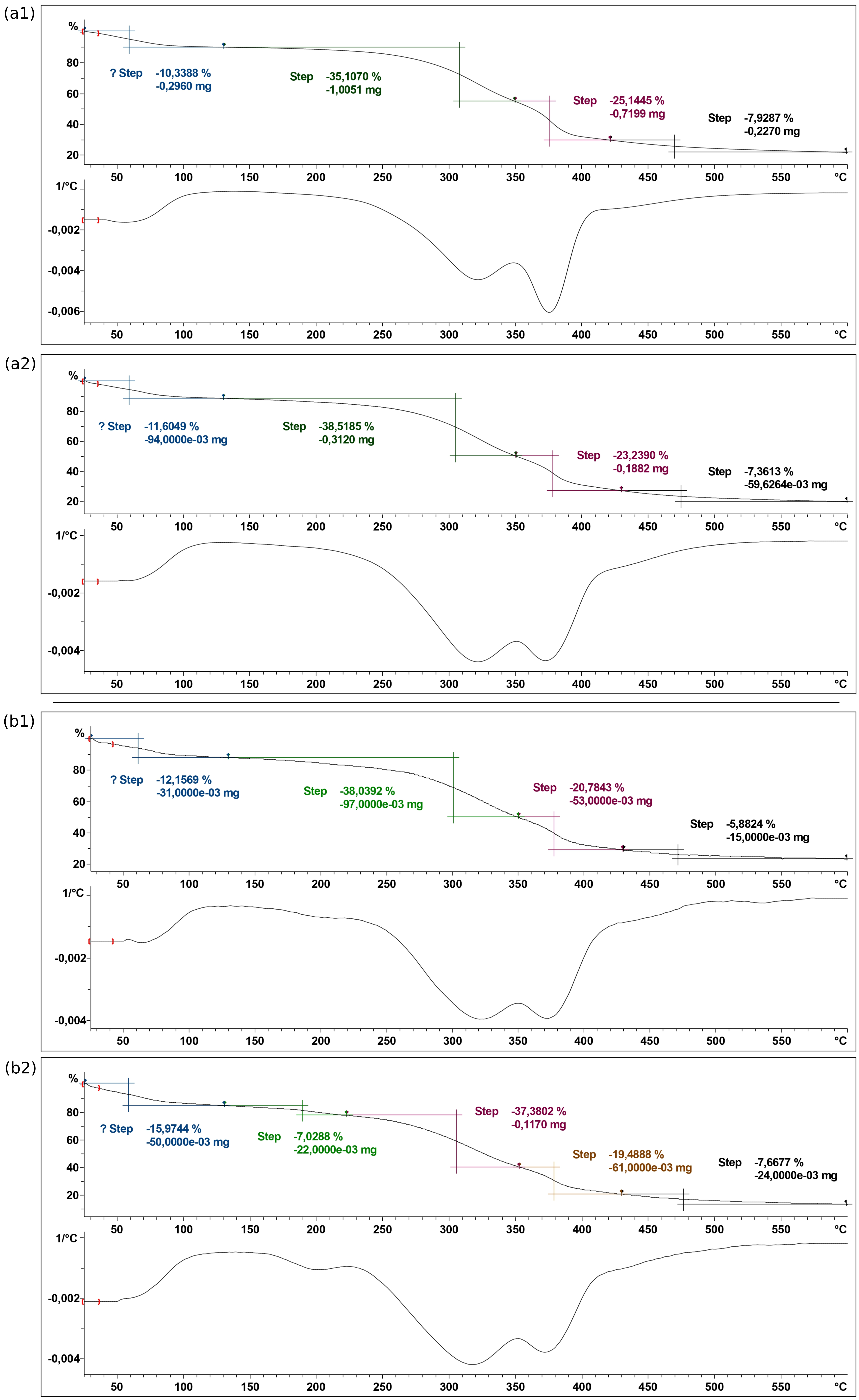

Supplement: Supplementary file 1 [file materials-17-05084-s001.zip › Figure S3.1.tiff]

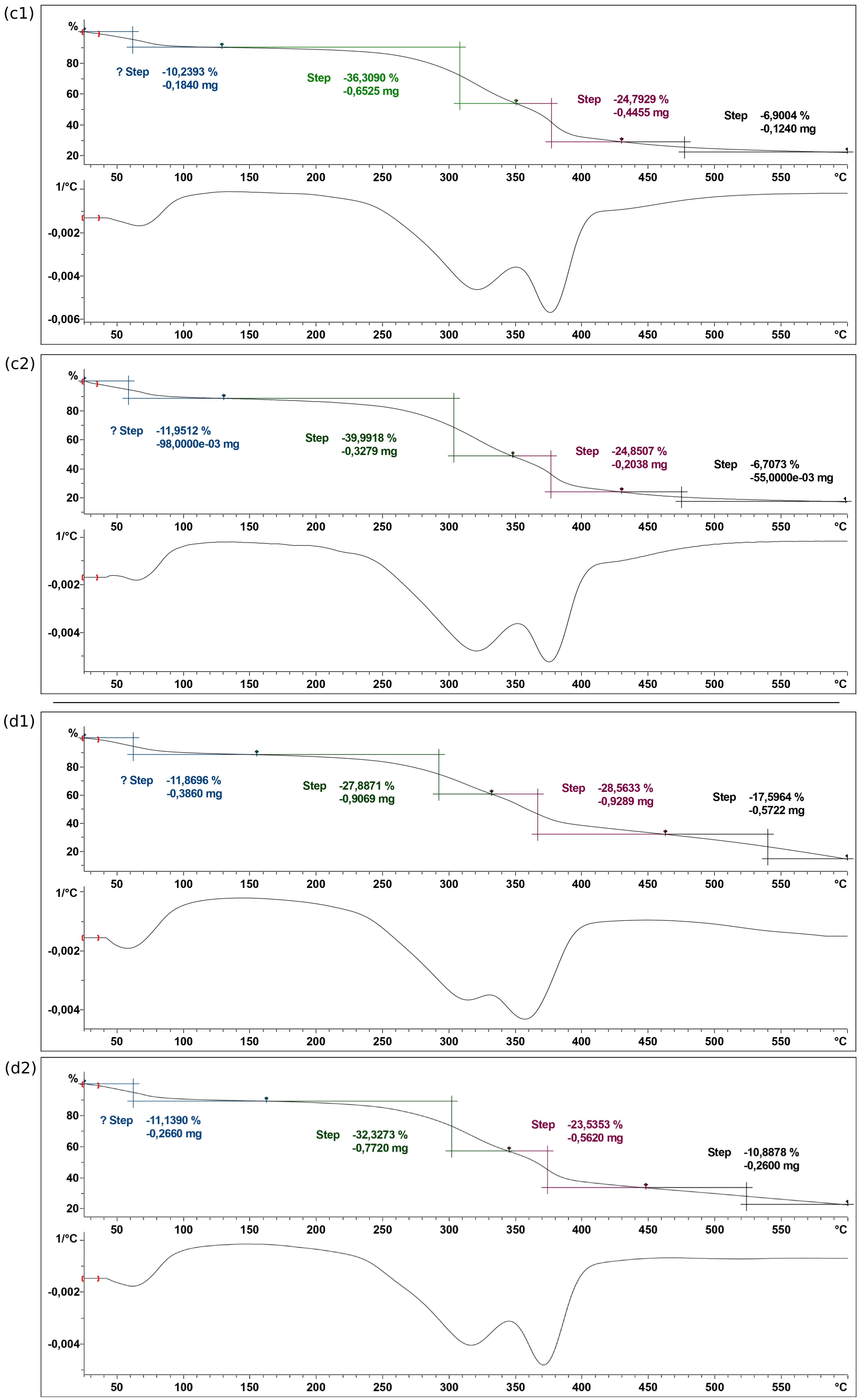

Supplement: Supplementary file 1 [file materials-17-05084-s001.zip › Figure S3.2.tiff]

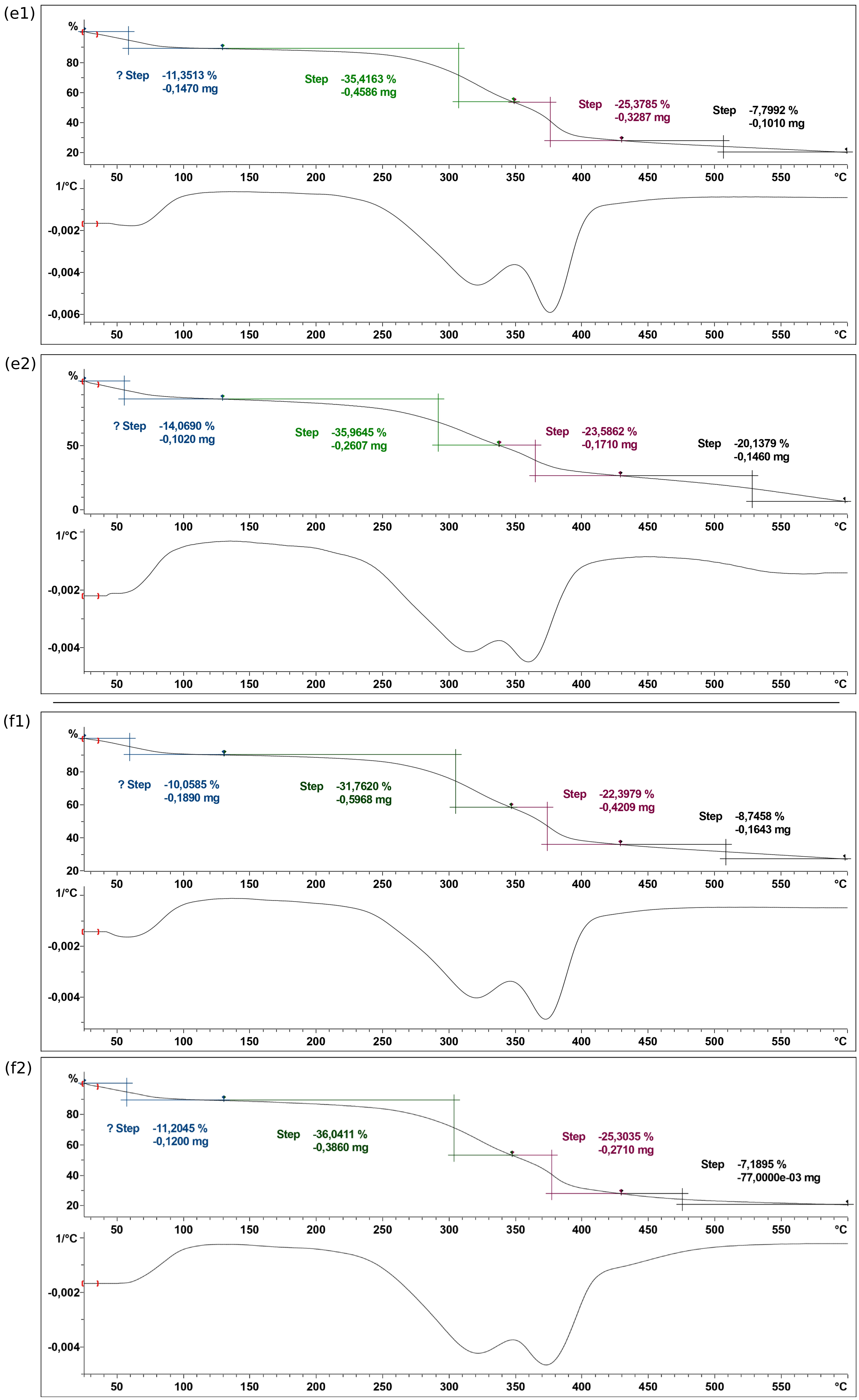

Supplement: Supplementary file 1 [file materials-17-05084-s001.zip › Figure S3.3.tiff]

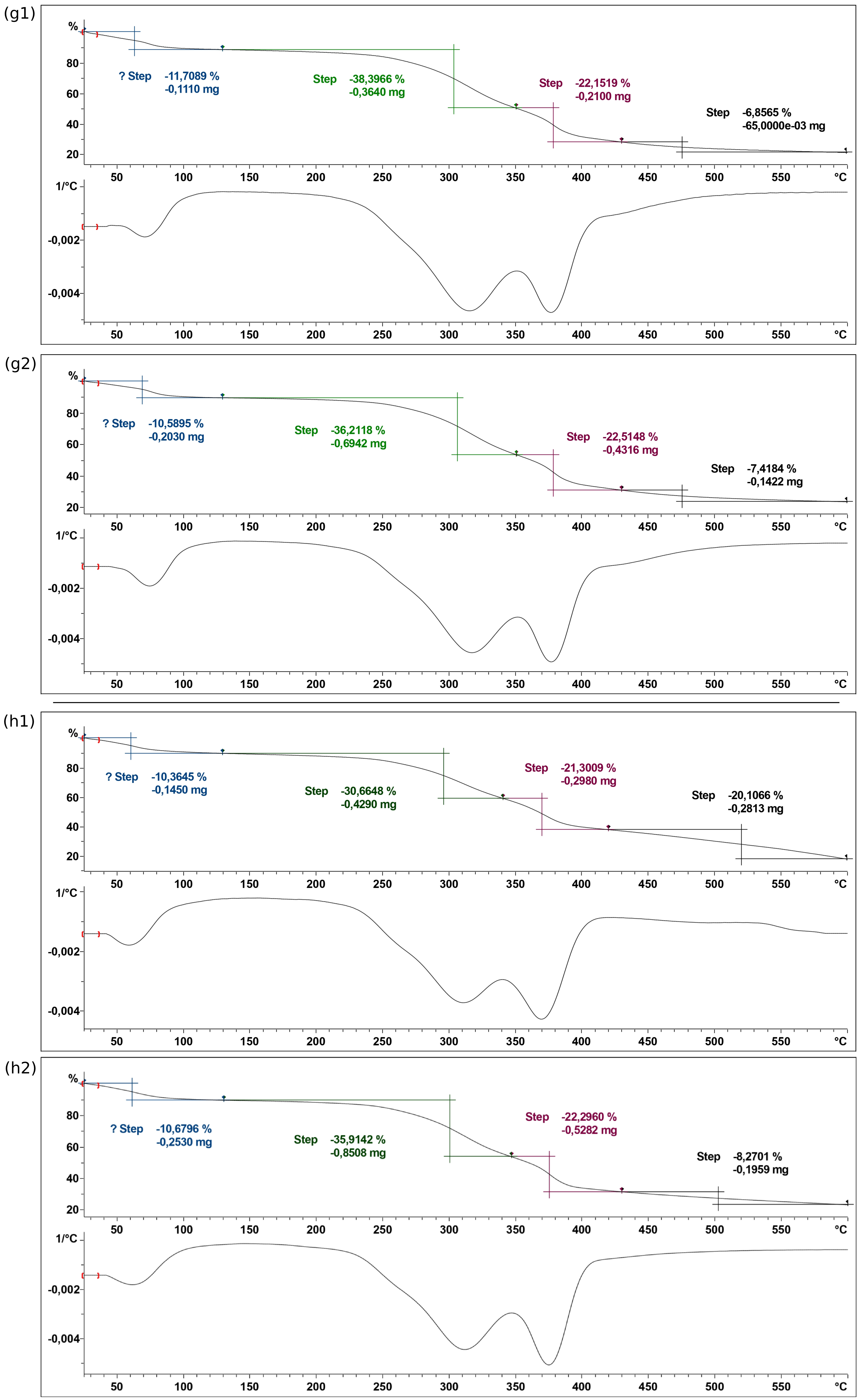

Supplement: Supplementary file 1 [file materials-17-05084-s001.zip › Figure S3.4.tiff]

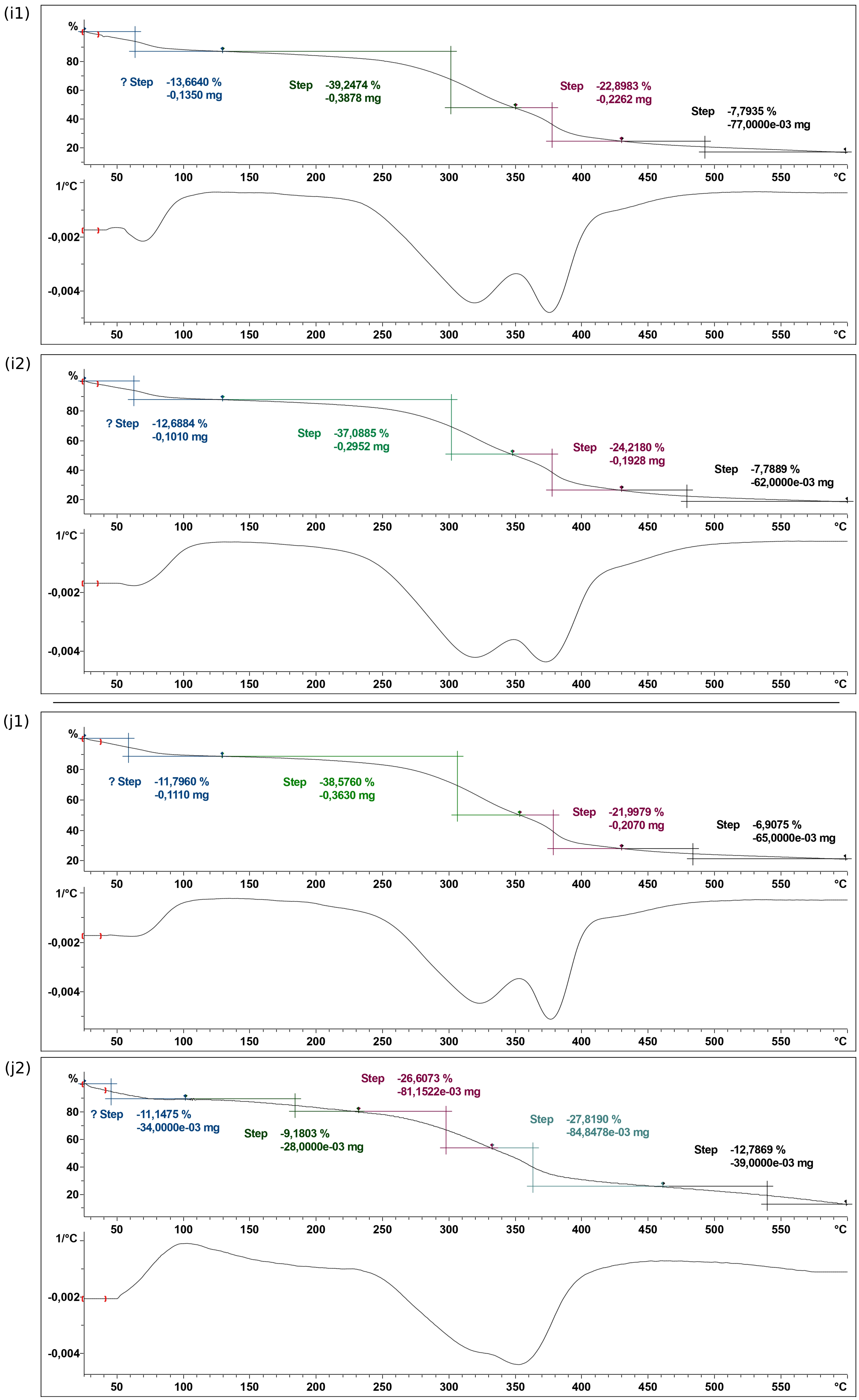

Supplement: Supplementary file 1 [file materials-17-05084-s001.zip › Figure S3.5.tiff]

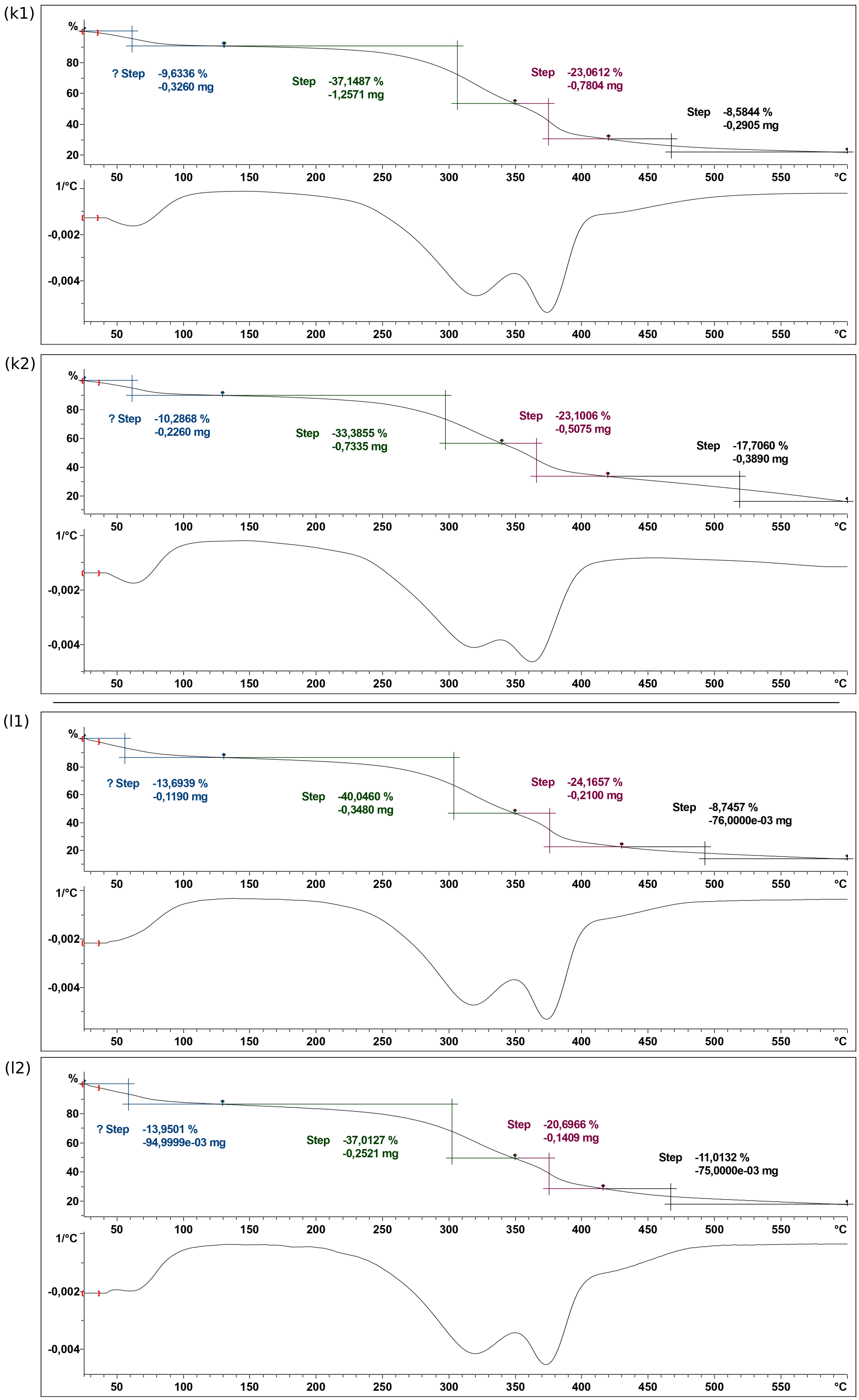

Supplement: Supplementary file 1 [file materials-17-05084-s001.zip › Figure S3.6.tiff]

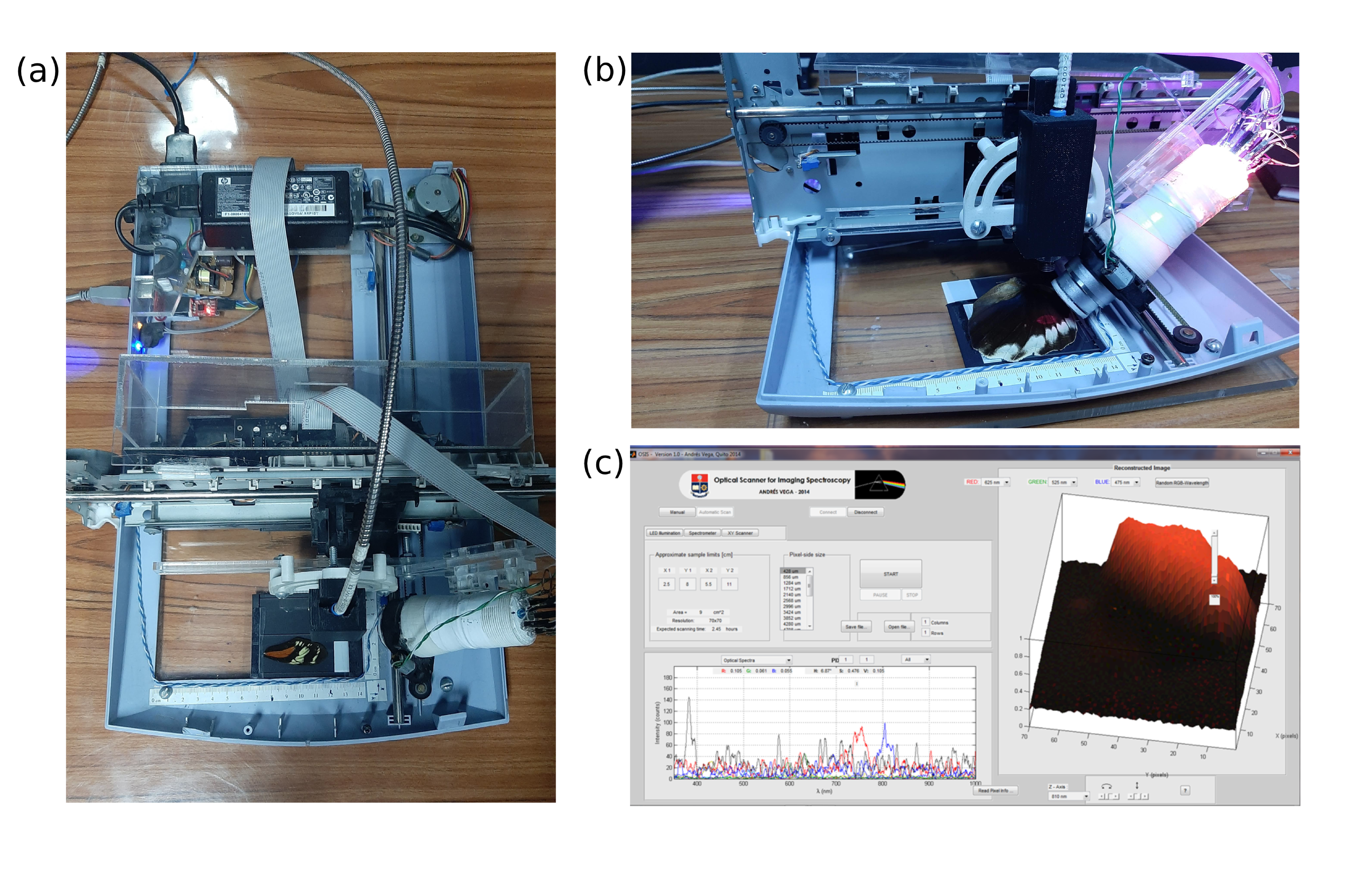

Supplement: Supplementary file 1 [file materials-17-05084-s001.zip › Figure S4.tiff]
